# Supplementary figures and images for: Viewing of abstract art follows a gist to survey gaze pattern over time regardless of broad categorical titles
Source: PLoS One. 2025 Jun 12;20(6):e0308591. doi: 10.1371/journal.pone.0308591 (PMC12161545; doi:10.1371/journal.pone.0308591)

**Supplementary Material**

**S1 Fig.** All in-house paintings created and used as stimuli are shown here.


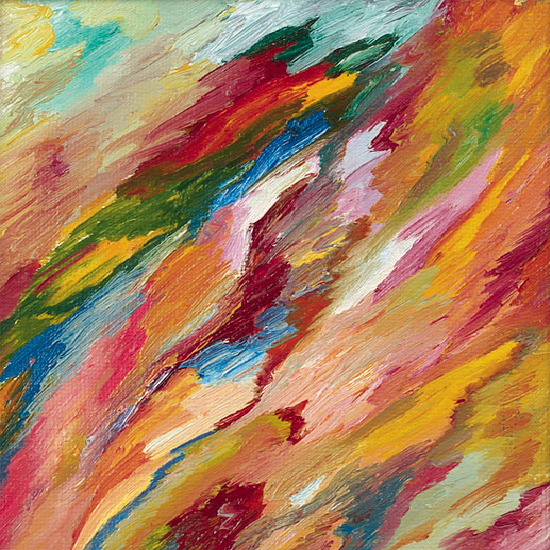

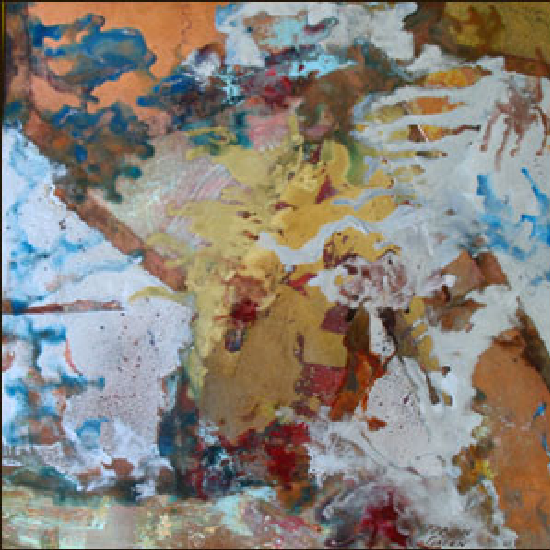

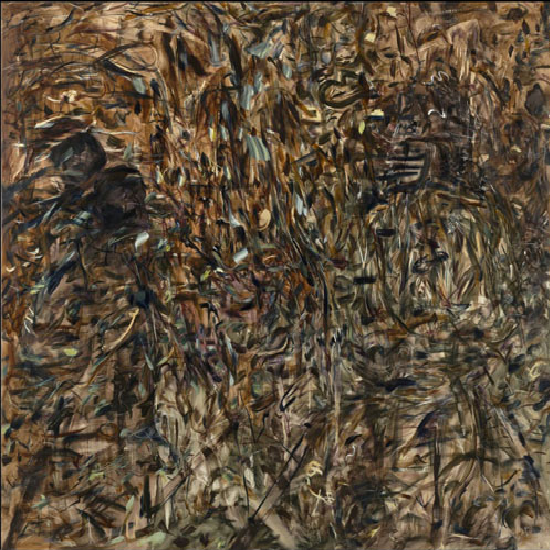

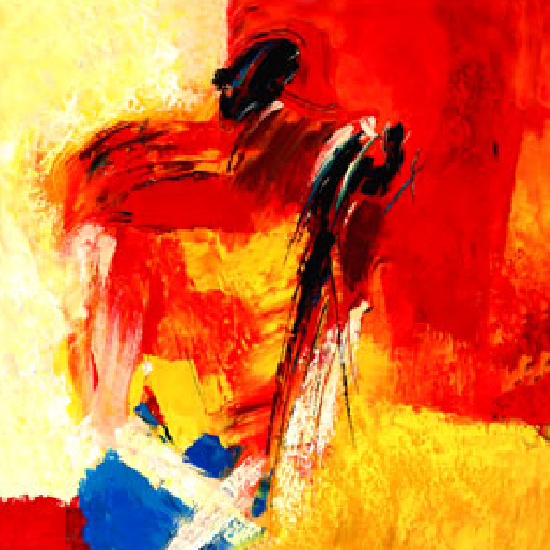

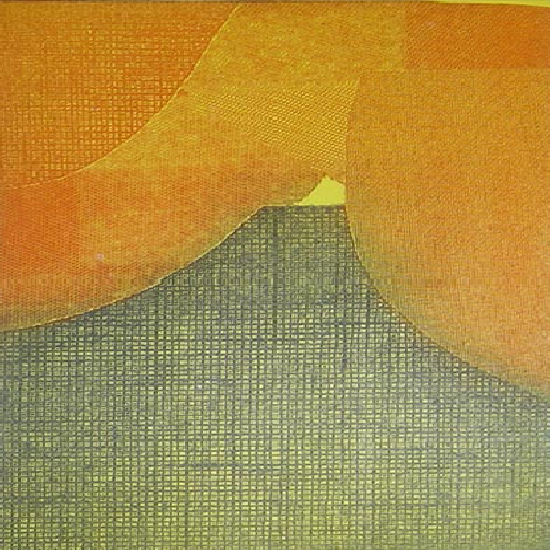

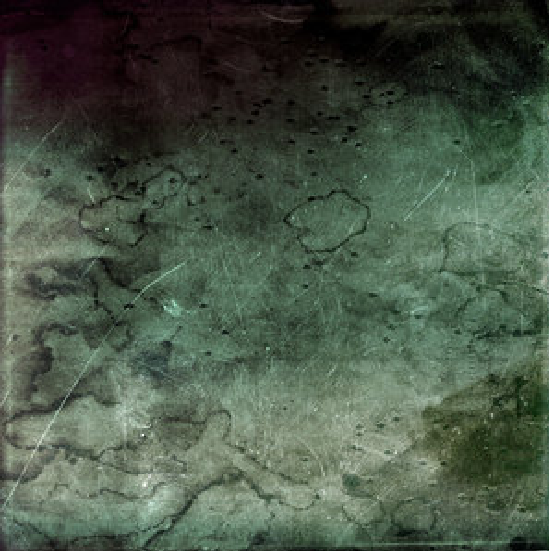

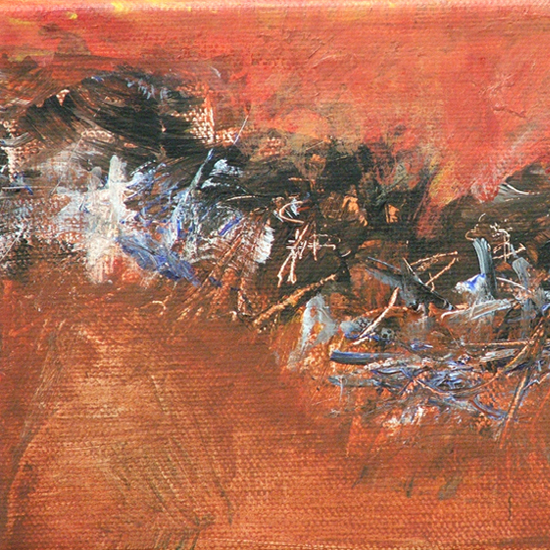

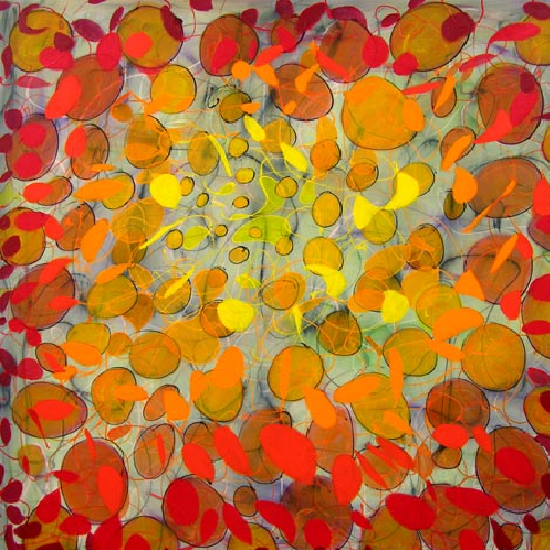

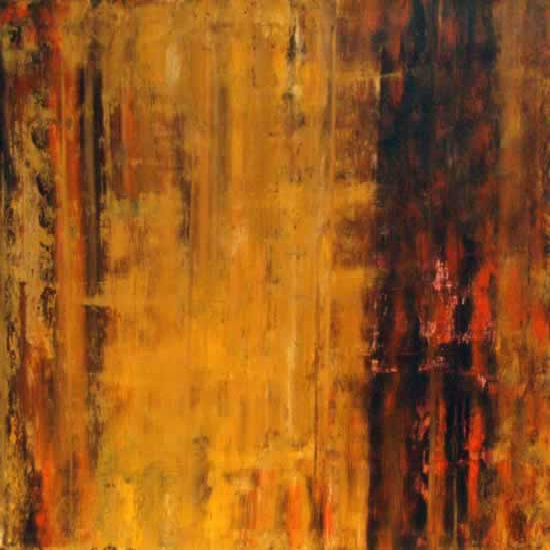

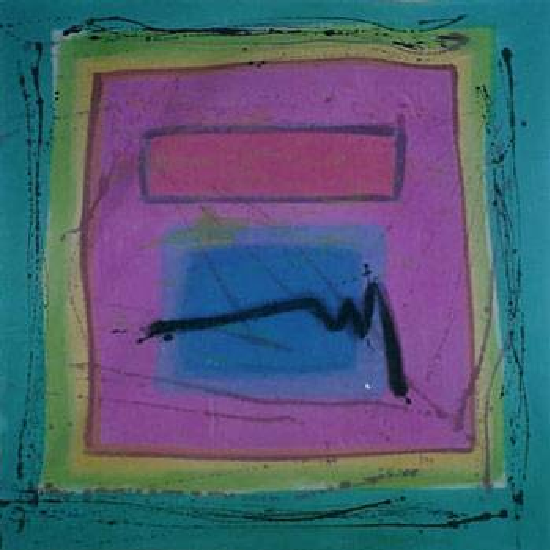

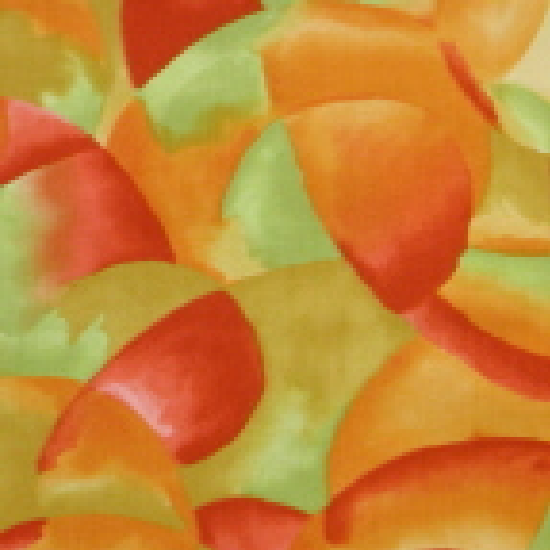

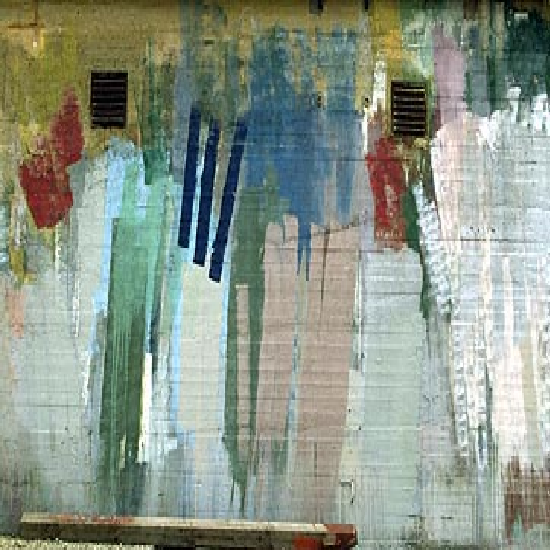

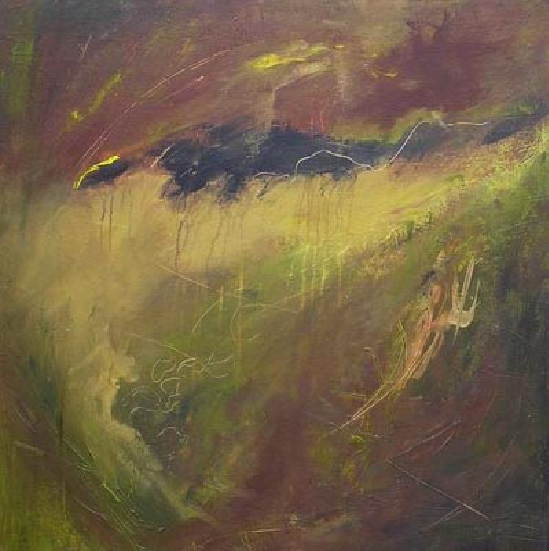

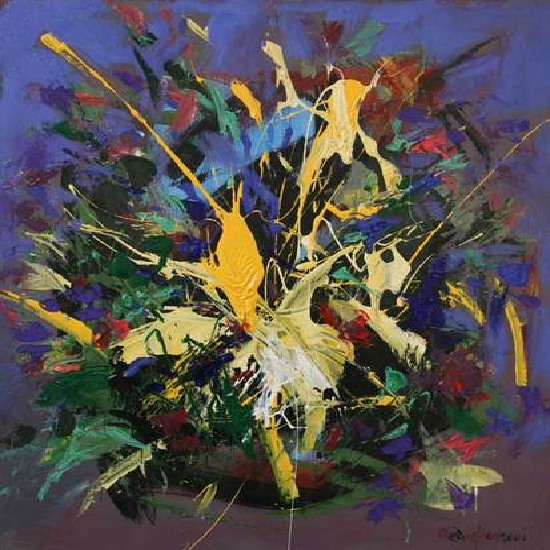

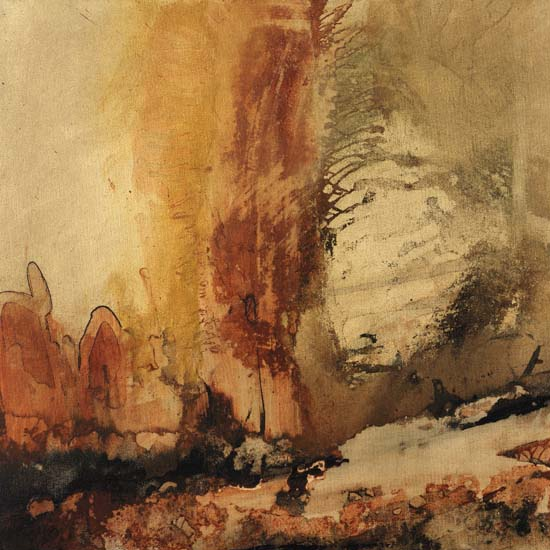

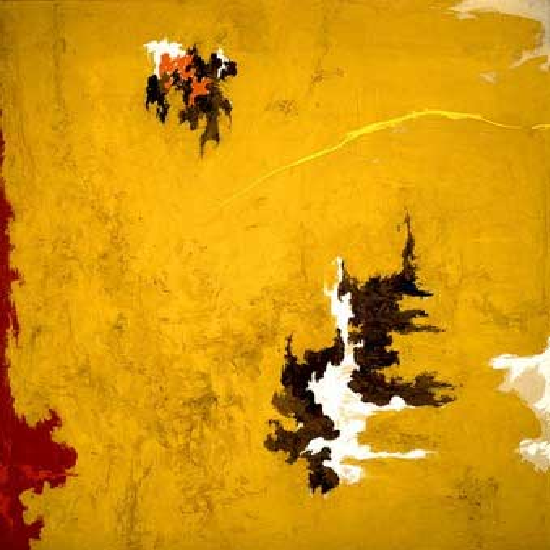

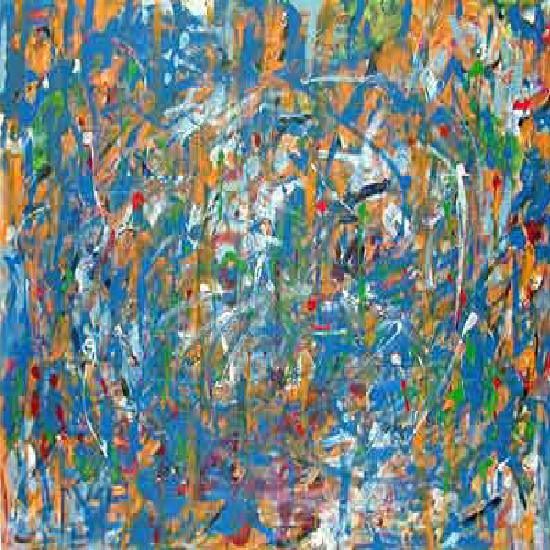

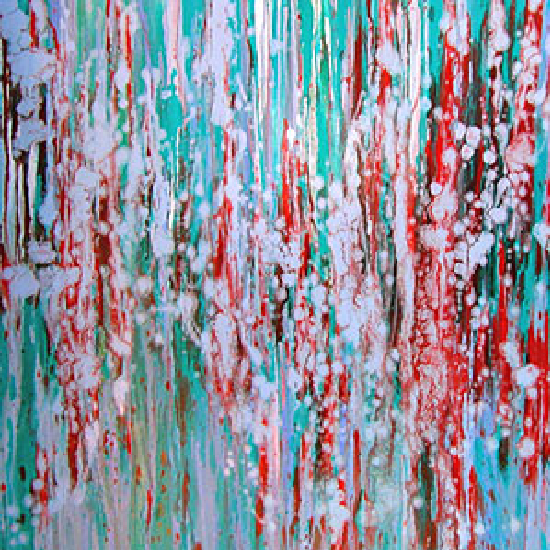

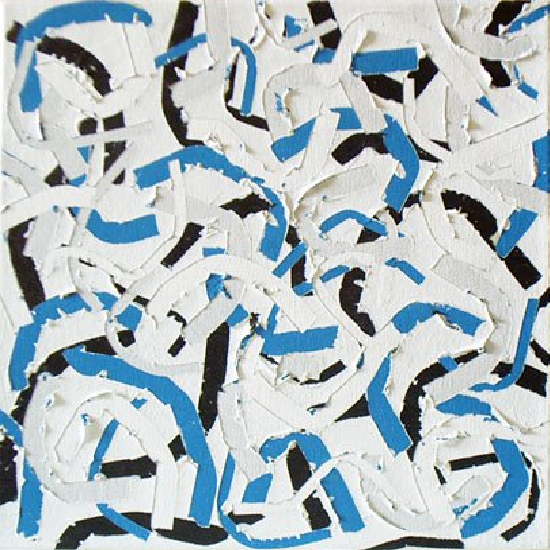

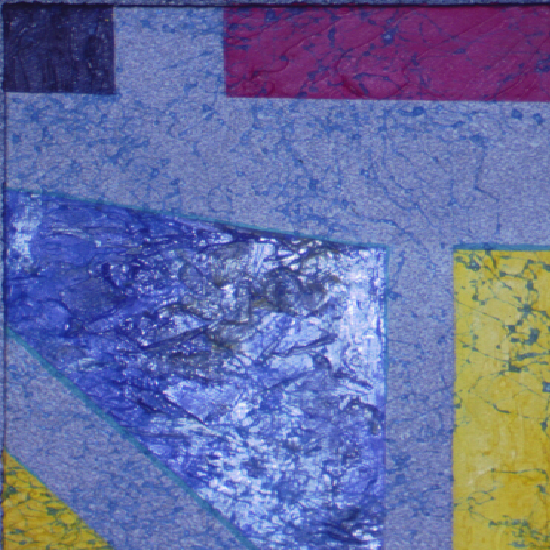

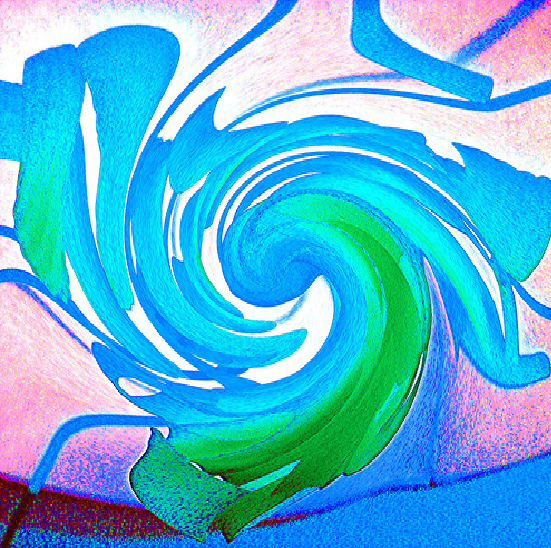

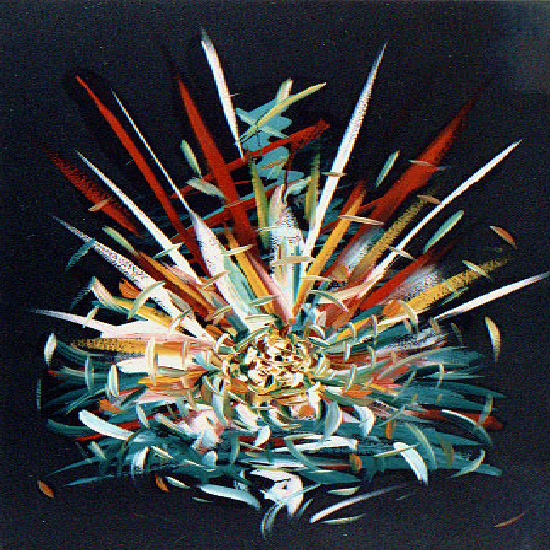

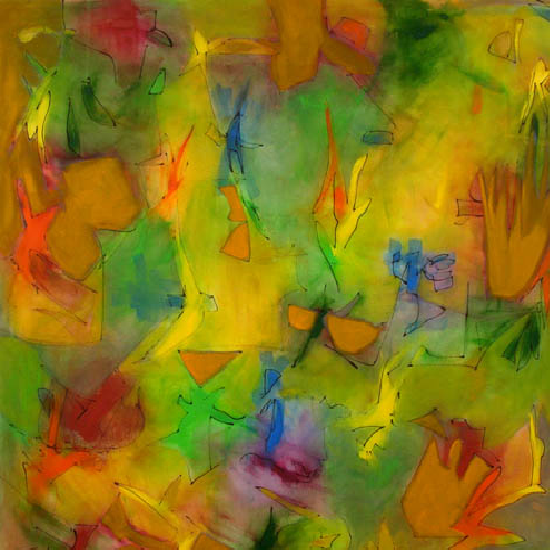

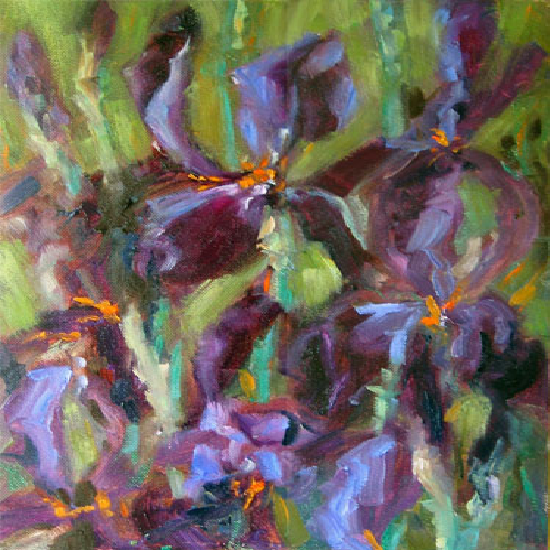

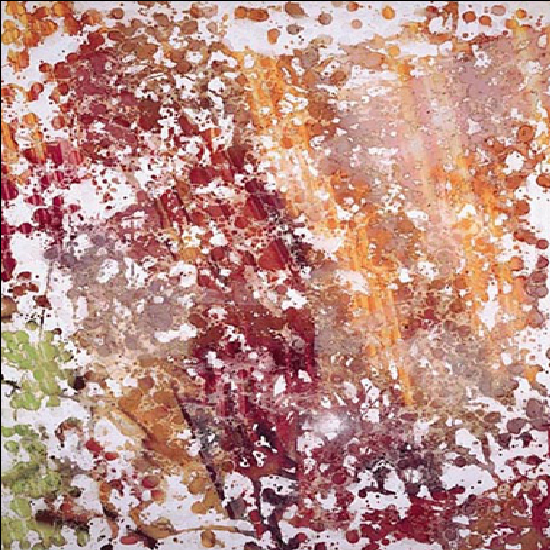

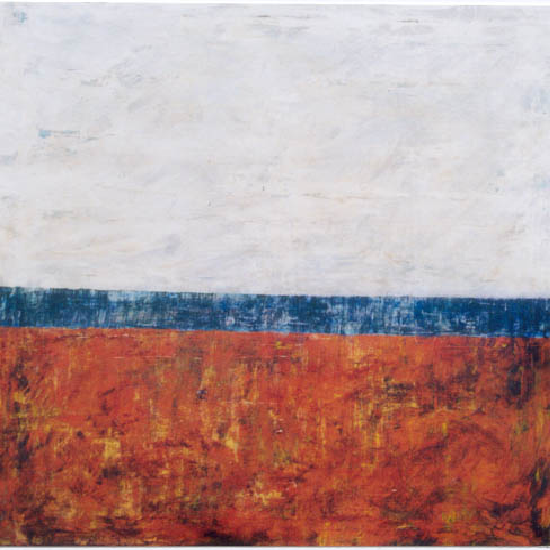

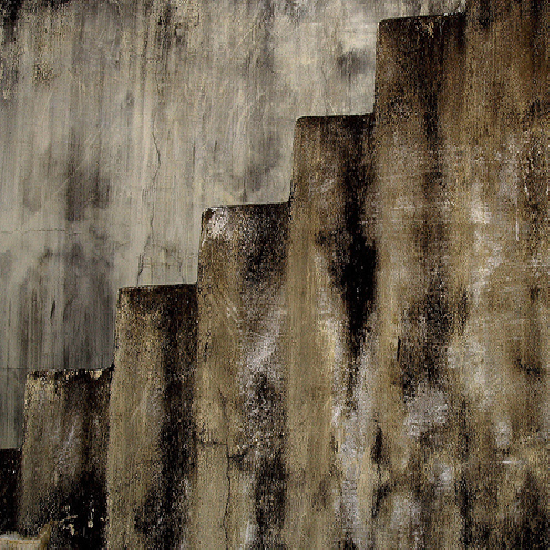

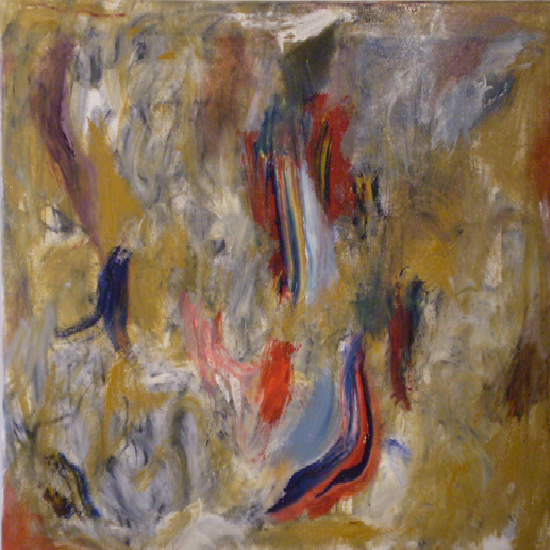

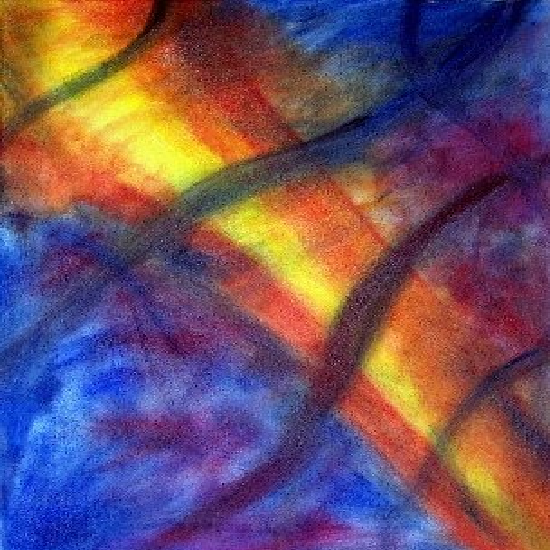

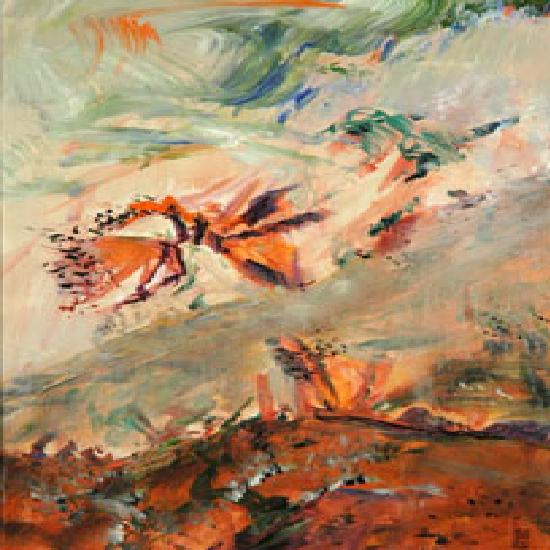

Supplement: S1 File — (DOCX) [file pone.0308591.s001.docx]
