# Supplementary material for: Viewing of abstract art follows a gist to survey gaze pattern over time regardless of broad categorical titles
Source: PLoS One. 2025 Jun 12;20(6):e0308591. doi: 10.1371/journal.pone.0308591 (PMC12161545; doi:10.1371/journal.pone.0308591)
Supplement: Attachment — Submitted filename: review_PONE.docx [file pone.0308591.s004.docx]

**Reviewer’s report**

In general, I believe the authors have addressed the issues raised by the reviewers. However, there is one further important point that I suggest to address.

The manuscript would benefit from a more in-depth discussion of the potential impact of individual differences in eye movement patterns while viewing artworks.

Recent research has highlighted this aspect. For example, Zangrossi et al. (2021) demonstrated that individual differences in viewing style (e.g., static vs. dynamic viewers) are stable across different image categories and even persist when participants are watching a blank screen. Specifically, static viewers exhibit fewer but longer fixations, whereas dynamic viewers display a more ambient viewing pattern characterized by a greater number of shorter fixations. A subsequent study by the same group (Celli et al., 2022) found that these viewing styles are associated with stable differences in resting-state brain activity.

Additionally, several studies have reported idiosyncratic eye movement profiles when individuals perform different oculomotor tasks (Poynter et al., 2013), including distinctive "oculomotor signatures" (Bargary et al., 2017; Kennedy et al., 2017).

Taken together, these findings suggest that, at least in part, the results presented in the manuscript may be influenced by interindividual differences, which should be considered in the discussion. This point is particularly critical for the present study since abstract artworks potentially leave more space to interindividual differences (i.e., endogenous processes guiding eye movements) as compared to more structured paintings (e.g., portraits, complex scenes, …).

**References**

- Bargary, G., Bosten, J. M., Goodbourn, P. T., Lawrance-Owen, A. J., Hogg, R. E., & Mollon, J. D. (2017). Individual differences in human eye movements: An oculomotor signature? *Vision Research*, *141*, 157–169. https://doi.org/10.1016/j.visres.2017.03.001
- Celli, M., Mazzonetto, I., Zangrossi, A., Bertoldo, A., Cona, G., & Corbetta, M. (2022). One-year-later spontaneous EEG features predict visual exploratory human phenotypes. *Communications Biology*, *5*(1), 1361. https://doi.org/10.1038/s42003-022-04294-9
- Kennedy, D. P., D’Onofrio, B. M., Quinn, P. D., Bölte, S., Lichtenstein, P., & Falck-Ytter, T. (2017). Genetic Influence on Eye Movements to Complex Scenes at Short Timescales. *Current Biology*, *27*(22), 3554-3560.e3. https://doi.org/10.1016/j.cub.2017.10.007
- Poynter, W., Barber, M., Inman, J., & Wiggins, C. (2013). Individuals exhibit idiosyncratic eye-movement behavior profiles across tasks. *Vision Research*, *89*, 32–38. https://doi.org/10.1016/j.visres.2013.07.002
- Zangrossi, A., Cona, G., Celli, M., Zorzi, M., & Corbetta, M. (2021). Visual exploration dynamics are low-dimensional and driven by intrinsic factors. *Communications Biology*, *4*(1), 1100. https://doi.org/10.1038/s42003-021-02608-x
