# Supplementary material for: Viewing of abstract art follows a gist to survey gaze pattern over time regardless of broad categorical titles
Source: PLoS One. 2025 Jun 12;20(6):e0308591. doi: 10.1371/journal.pone.0308591 (PMC12161545; doi:10.1371/journal.pone.0308591)
Supplement: Attachment — Submitted filename: Response to Reviewers.docx [file pone.0308591.s006.docx]

**Reviewer comments:**
1. Your article is well-revised and meets a high standard for publication. I have a minor suggestion: consider including a visualization of raw eye movement data overlaid on the abstract art, showing fixation points and saccades for a representative 10-second segment from both experiments. This would help the audience intuitively grasp the gaze patterns before presenting the bar graphs. Additionally, a brief explanation in the results section would enhance clarity and provide better context for the statistical summaries.

**Response:**

While we thank the reviewer for their comment and suggestion. We did consider the addition of the visualizations requested to the manuscript and have elected include examples of these as a new figure with supporting text. To provide a context for data analysis and improve clarity these have been added to the manuscript at the beginning of the Results section. As the art works shown to viewers differed across trial then aggregating scan paths across trials would not make sense. Nor would aggregating scan paths across participant. This leaves the option to include single trial example images of fixation points and saccades scan paths for individual participants from each experiment and condition. The result of this is shown below and has been incorporated into the text as follows:

Addition to the main body of the text of Results section:

“To help the reader visualize the eye movements we recorded example plots of recorded scan paths. These are shown in Fig 2 overlaid upon the abstract artwork that evoked that scan path response. Each artwork and scan path shown is a single trial from different participants. Those on the left are from Experiment 1 (labelled for each participant as: “Landscape”, “Portrait” and “Abstract” from top to bottom) and those on the right are from Experiment 2 (labelled “Still Life”, “Action” and “Abstract” from top to bottom). Plots show fixation locations as blue circles along with the start and end points of each saccade (with a joining straight yellow line). Fixation durations are represented by circle diameter with wider ones indicating longer durations as shown by the number next to each circle which shows the actual duration in milliseconds. The location of the saccade in the scan path sequence is given the yellow number towards the end of each line e.g., 11 would mean the 11^th^ saccade in that scan path sequence for that trial.”

Additional Figure legend in text of manuscript:

**“Fig 2: shows example scan paths for six abstract art works for six different participants.** The three on the left are from Experiment 1 (labelled for each participant as: “Landscape”, “Portrait” and “Abstract” from top to bottom) and those on the right are from Experiment 2 (labelled “Still Life”, “Action” and “Abstract” from top to bottom). Fixations are plotted as pale blue circles, the center of each is the fixation location while their diameter represents their duration which is also given by the number (in milliseconds) next to each circle. Saccades are shown as straight yellow lines between their start and end points with the yellow number indicating the point at which each saccade as executed in the path sequence (i.e., 1 is the first saccade, 2 is the second and so on).”

The Figure itself:

**Reviewer's report:**

In general, I believe the authors have addressed the issues raised by the reviewers. However, there is one further important point that I suggest to address.

The manuscript would benefit from a more in-depth discussion of the potential impact of individual differences in eye movement patterns while viewing artworks.

Recent research has highlighted this aspect. For example, Zangrossi et al. (2021) demonstrated that individual differences in viewing style (e.g., static vs. dynamic viewers) are stable across different image categories and even persist when participants are watching a blank screen. Specifically, static viewers exhibit fewer but longer fixations, whereas dynamic viewers display a more ambient viewing pattern characterized by a greater number of shorter fixations. A subsequent study by the same group (Celli et al., 2022) found that these viewing styles are associated with stable differences in resting-state brain activity.

Additionally, several studies have reported idiosyncratic eye movement profiles when individuals perform different oculomotor tasks (Poynter et al., 2013), including distinctive "oculomotor signatures" (Bargary et al., 2017; Kennedy et al., 2017).

Taken together, these findings suggest that, at least in part, the results presented in the manuscript may be influenced by interindividual differences, which should be considered in the discussion. This point is particularly critical for the present study since abstract artworks potentially leave more space to interindividual differences (i.e., endogenous processes guiding eye movements) as compared to more structured paintings (e.g., portraits, complex scenes, ‚Ä¶).

References

Bargary, G., Bosten, J. M., Goodbourn, P. T., Lawrance-Owen, A. J., Hogg, R. E., & Mollon, J. D. (2017). Individual differences in human eye movements: An oculomotor signature? Vision Research, 141, 157-169. https://doi.org/10.1016/j.visres.2017.03.001

Celli, M., Mazzonetto, I., Zangrossi, A., Bertoldo, A., Cona, G., & Corbetta, M. (2022). One-year-later spontaneous EEG features predict visual exploratory human phenotypes. Communications Biology, 5(1), 1361. https://doi.org/10.1038/s42003-022-04294-9

Kennedy, D. P., D'Onofrio, B. M., Quinn, P. D., B√∂lte, S., Lichtenstein, P., & Falck-Ytter, T. (2017). Genetic Influence on Eye Movements to Complex Scenes at Short Timescales. Current Biology, 27(22), 3554-3560.e3. https://doi.org/10.1016/j.cub.2017.10.007

Poynter, W., Barber, M., Inman, J., & Wiggins, C. (2013). Individuals exhibit idiosyncratic eye-movement behavior profiles across tasks. Vision Research, 89, 32-38. https://doi.org/10.1016/j.visres.2013.07.002

Zangrossi, A., Cona, G., Celli, M., Zorzi, M., & Corbetta, M. (2021). Visual exploration dynamics are low-dimensional and driven by intrinsic factors. Communications Biology, 4(1), 1100. https://doi.org/10.1038/s42003-021-02608-x

**Response:**

Many thanks for the suggestion and for the additional references. As a result of reading these the additional text has been added to the Discussion (all additional numbered references have been added to the reference list):

A further consideration in the control of gaze when viewing artworks is a potential role of stable individual differences in viewing behavior and the shift from the Gist-Ambient to the Survey-Focal phase of art viewing. Measures of oculomotor behavior exhibit stable characteristics that can be captured with common descriptive statistics, but they also reveal a wide range in individual responses. This can be seen in large population studies of saccades, anti-saccades and smooth pursuit (1–4). Despite these dissimilarities, recent research has identified stable gaze patterns within individuals. Across a range of diverse tasks individuals produce similar eye movement behaviours such as fixation numbers, fixation durations, and saccade amplitudes (5–10), with a similar consistency also shown in measures of micro-saccadic eye movement behavior such as fixation extent, micro-saccade number and amplitude (11). Recently Bargary et al (12) reported reliable individual pro- and anti- saccade and smooth pursuit eye movement patterns across task and test-retest sessions with eye movement parameters that correlated well within eye movement type. These have been likened to an oculomotor signature (12) and have been related to differences in personality traits (13) and genetic influences (14). Poynter et al (11) identified strong correlations across six eye movement metrics (e.g., fixation rate, duration and size, saccade amplitude, microsaccade rate and amplitude) that were captured by a single underlying latent factor. Zangrossi et al (15) also reported that viewing behavior dynamics of a large sample of participants in response to a wide range of real-world natural scenes was explained well by very few latent variables with only three components explaining about 60% of eye movement dynamics (those that more reflect how or when people look: fixation duration, and number, gaze step direction and amplitude of saccade). Unlike gaze dynamics analysis of the spatial distribution of fixations (where people look: density map, semantic map and saliency map) show a dependency of gaze distribution on image meaning and points of saliency. The low dimensionality of gaze dynamics led Zangrossi et al (15) to identify individual viewing styles into the two stable types of static and dynamic viewers, that reflective automatic intrinsic, endogenous preferences that are executed relatively independently of differences in the external visual environment and remain stable when participants view a blank screen. These viewing styles have been linked to persistent differences in resting-state EEG brain activity (16) shown in static and dynamic viewers tested one year after behavioral experiments reported by Zangrossi et al (15). These differences in resting-state have been linked to higher cortical inhibition and focus on internal processing for static viewers while dynamic viewers have a profile more biased toward cortical excitation and external processing. This interpretation is bolstered by behavioral outcome measures with static viewers showing slightly stronger visual working memory and dynamic viewers showing weaker inhibition of salient but non-relevant stimuli. These stable individual differences in eye movement control and viewing behaviours are likely to also play a role in gaze patterns while viewing artworks and in the eye movement outcomes reported here. This is especially likely when the stimuli viewed were abstract artworks which may encourage eye movements that reflect an individual’s idiosyncratic endogenous internally driven viewing style rather than being influenced by the artwork’s semantic content, something that would be found in more structured paintings such as portraits or landscapes. This is something that would be fruitful to more thoroughly explore in future studies in this area.
